# Influence of Aquatic Therapy in Children and Youth with Cerebral Palsy: A Qualitative Case Study in a Special Education School

**DOI:** 10.3390/ijerph17103690

**Published:** 2020-05-23

**Authors:** Elisa Muñoz-Blanco, Javier Merino-Andrés, Beatriz Aguilar-Soto, Yolanda Castillo García, Marta Puente-Villalba, Jorge Pérez-Corrales, Javier Güeita-Rodríguez

**Affiliations:** 1 Department of Physical Therapy, Faculty of Medicine, CEU-San Pablo University, 28003 Madrid, Spain; Elisa.munozblanco@ceu.es; 2 Research and Science Committee of Worldwide Aquatic Bodywork Association (WABA), 6802 Monteceneri, Switzerland; 3 Faculty of Physical Therapy and Nursing, Universidad de Castilla La Mancha, 45071 Toledo, Spain; Javier.Merino@uclm.es; 4 PedPT Research Lab, Grupo de Investigación de Fisioterapia en Toledo (GIFTO), 45071 Toledo, Spain; 5 Riverside Care and Support Vaughan House, Guilford GU1 4HD, UK; Bea.aguilarsoto@riverside.org.uk; 6 CCEE Nª Sra del Prado APACE Talavera, 45600 Toledo, Spain; Yoal95@hotmail.com; 7 Escuela De Actividades En La Naturaleza (ECUNATUR), 45600 Toledo, Spain; Ecunatur@gmail.com; 8 Department of Physical Therapy, Occupational Therapy, Rehabilitation and Physical Medicine, Rey Juan Carlos University, Alcorcón, 28922 Madrid, Spain; Jorge.perez@urjc.es; 9 Research Group of Humanities and Qualitative Research in Health Science, Rey Juan Carlos University (Hum&QRinHS), Alcorcón, 28922 Madrid, Spain

**Keywords:** cerebral palsy, special education, physical therapy modalities, qualitative research

## Abstract

Cerebral palsy results in the progressive loss of motor functions, with a negative impact on daily activities and participation. Despite the well described benefits of aquatic therapy in children, little is known about the effects of the same in school settings. This study aimed to describe the experience of children and youth with cerebral palsy participating in an aquatic therapy program within a special education school considering their educational and therapeutic perspectives. A qualitative descriptive case study with embedded units was developed, comprising 27 participants. This study employed purposeful sampling to include children and youth with cerebral palsy from the Asociación Ayuda a la Paralisis Cerebral (APACE) special education school, together with their parents, the special education teachers, and health care professionals. Data were collected via non-participant observation, semi-structured and informal interviews, focus groups, and researcher field notes. A thematic analysis was conducted, revealing the following themes: (a) the connection with the environment; (b) postural improvements and mobility; (c) the opportunity to perform tasks; (d) learning and transfer. A motivating environment leads to physical, cognitive and social benefits, both at school and in the home. Aquatic therapy was viewed as a means for learning and participation. These findings may enhance understanding regarding the potential benefits of implementing multidisciplinary aquatic therapy programs in specialist school settings.

## 1. Introduction

Cerebral palsy (CP) is a lifelong disorder that produces a variety of sensory-motor, cognitive, communication, and behavioral disorders that influence children’s activities and participation [1]. Rehabilitation is a complex process that seeks to promote the highest possible levels of participation and quality of life, for both the child and the family. Often, these children cannot attend regular schools and therefore require special education. Students with CP have major needs at school, and health professionals have a role to play in advocating, educating, and supporting students at school [2]. Joint educational and rehabilitative programs should be person-centered, considering all dimensions, while involving the child, the family, life events, and the environment, in order to address the numerous problems in a holistic manner [2].

There are significant differences between the level of participation and performance of activities at school among typical students and students with CP [3]. Mei et al. found that the activities and participation of children with CP are mainly related to early learning tasks, communication, mobility, and interactions, based on the experiences of parents. According to the previously mentioned authors, therapists must seek to overcome the potential negative impact of the illness on participation levels, by addressing physical difficulties, as well as numerous social and environmental factors, such as communication, support, or environmental attitudes [4]. These findings emphasize the continuing need to provide rehabilitation interventions aimed at overcoming learning and environmental barriers to support inclusive education [3]. An inclusive school culture is crucial for students with CP. Their rehabilitation and education environments must prioritize the promotion of an open and positive school culture built around inclusive problem-solving practices. Thus, school staff, health professionals, families, and students can work together to improve the student experience [2].

Children and youth with CP who undergo rehabilitation in specialized schools may respond differently to structured exercise programs, particularly with regard psychosocial outcomes such as mental health, and academic and social development [5]. The inclusion of aquatic therapy (AT) programs in schools may be beneficial as aquatic exercise can provide a fun and motivating form of physical activity, supporting the physical, social, and emotional well-being of children and youth with CP [6,7,8]. In addition, water exercise is among the most common physical activity modality chosen by children with CP and their parents [9], often representing the first choice in addition to regular therapy [10]. Often, AT is more conducive to independent activities and participation compared to many conventional land-based therapies. A number of therapeutic effects have been demonstrated for AT, along with relevant clinical effects. This is reflected in the results of systematic reviews on children with CP, with improvements of gross motor skills, walking endurance and gait efficiency related to systemic cardiorespiratory adaptations in adolescents with CP [6,7,8]. Furthermore, aquatic exercise has shown to be feasible with minimal adverse effects [8].

The Aquatic Therapy Core Sets (CSs) describe the aquatic therapy profile of children and youth with neurological disorders [11]. The development of CSs for the specific health care context of AT assists clinicians in identifying relevant categories to describe function and appropriate measurement tools to use in practice and research related to AT [12]. However, these CSs did not include the educational environment in the context where these activities take place. This study is an attempt to research how to operationalize the included AT CSs categories in school settings. The aim of this study was to describe the experience of children with cerebral palsy participating in a therapeutic aquatic program within a special education school, as well as that of their parents and the education and therapy professionals.

## 2. Materials and Methods

Qualitative studies are useful for understanding the beliefs, values, and motivations that underlie individual health behaviors [13]. Furthermore, qualitative studies have been used to research the experience of parents of children and youth with CP to identify a list of relevant intervention categories for AT [14], as well as issues regarding their experiences of evidence-based assessment practices [15] and the participation in community-based physical activity [16] or the barriers and facilitators for physical activity in special education [5].

### 2.1. Design

A qualitative case study with embedded units was developed [13,17,18,19]. These units comprise different participants, contexts, places, and moments, connected by the phenomenon under study [17,20]. In this study, the phenomenon under study was the influence of the application of AT in children and youth with CP in specialist schools, as experienced by different participants, (including students, parents, and other participants in their therapeutic and educational context, such as health care professionals and special education teachers). This study encompasses the family and the health context, and it appears in different places such as school rooms, homes, and swimming pool. For this reason, a qualitative descriptive case study with embedded units was conducted [17,19]. A case study may be formed of different units, all of which help to describe a complex phenomenon. These units may be different participants, from different contexts and places who are only connected by the phenomenon under study [17,20]. A case study design should be considered when: (a) the focus of the study is to answer ‘how’ and ‘why’ questions; (b) you cannot manipulate the behavior of those involved in the study; and (c) one wants to cover contextual conditions because he or she believes they are relevant to the phenomenon under study [18].

### 2.2. Context

The AT intervention took place at a pool located in the APACE special education school (Toledo, Spain, https://www.apacetalavera.es/centro-educacion-especial/). This AT treatment was part of the special education programs of the APACE-Talavera Association, which also includes an early intervention center, an occupational center, and a day center. The main goal of this AT program was to enhance the potential cognitive, sensorimotor, and social improvements among children and youth with CP, as a feasible alternative to conventional physical therapy. The interventions performed by aquatic therapists included Watsu, Craniosacral Therapy in Water and Water Specific Therapy-Halliwick. Individual AT sessions were performed by the same physical therapist, twice a week, with an average duration of 45 min, depending on age.

### 2.3. Participants

We included participants who could provide information regarding the phenomenon under study. As the AT intervention was framed within the rehabilitation program of a special education school, we included all those who participated in the program and who fulfilled the inclusion criteria.

Thus, the inclusion criteria for the participants in this case study were as follows:

(a) Family context: children and youth with a diagnosis of CP, between the ages of 3 and 21 (the age range for the school), who at the time of the study had been receiving aquatic therapy for at least one year and who signed the informed consent; their parents, who signed the informed consent.
(b) Educational and therapy contexts: health care and education professionals taking care of the children involved in AT at the time of the study, members of the teaching or rehabilitation staff, and who signed the informed consent.

The student exclusion criteria were: having an absenteeism rate of over two weeks for health reasons in the previous school year, not being able to verbally express themselves fluently, requiring communication aids, and refusing to participate in the study.

### 2.4. Sampling Strategies

A purposeful sampling strategy was employed [19,21] to deliberately select children and youth who participated in the AT program, together with their parents, teachers and therapists. Thus, 28 families who were participating in the AT program of the APACE school at the time of the study were contacted by sending them a letter and form. Ultimately, 14 families accepted to participate in the study, who fulfilled the inclusion criteria and whose parents granted consent for their participation. In addition, four teachers and four therapists who had worked with the children included in the study were invited to participate in this study. Finally, two teachers and three therapists agreed to participate. Thus, 27 participants were included in the final sample and there were no dropouts (Table 1).

### 2.5. Recruitment Procedure

The data collection consisted of two phases. In the first phase, non-participant observations and informal interviews were conducted. In the second phase, in-depth interviews and focus groups were conducted. The researchers explained the purpose and design of the study to the school principal and students during an initial face-to-face contact session. Separate information sessions were held for students, their legal guardians, and the health and education professionals. During these meetings, the study was described and questions were answered. Finally, participants were invited to participate in the study. All the informative sessions were held at the APACE school.

### 2.6. Research Team and Reflexivity

The research team was comprised of seven individuals (four women and three men). Three members were physical therapists, in addition there was a speech therapist, an occupational therapist, a social worker and a teacher. Four members of the team had clinical experience in pediatrics. Three researchers had prior experience in qualitative design studies. Prior to the study, the researchers’ positioning was established according to their previous experience and their motivations [19,22].

### 2.7. Data Collection

The aim of this case study was to obtain an in-depth and multi-perspective, holistic understanding on the phenomenon of interest, which implied the need for multiple data sources and multiple data collection instruments (Table 2) [20]. The data were collected through non-participant observation, informal interviews, semi-structured interviews, focus group and researcher field notes. This data gathered information on: (a) the meaning of AT and prior experiences, (b) the influence of AT within the school, (c) CP factors for which AT is most useful, and (d) difficulties implementing AT. A series of questions was established for each unit of analysis. (Supplemental Materials Tables S1–S3).

#### 2.7.1. Non-Participant Observation and Informal Interviews

Bearing in mind that most children and youth were cognitively impaired, together with the difficulty of maintaining a fluid communication process, data collection began with non-participant observation. According to this method, the researcher approaches the participants in their own environment, instead of vice versa. The observation was aimed at analyzing the impact of the AT intervention on the performance of the children’s functions and their participation in their educational environment. The researcher had no relationship with the observed group, attempting to collect observational field notes in a systematic and discrete manner. The settings for the observations were: (a) the designated therapy areas (swimming pool, speech therapy and occupational therapy work rooms); (b) the communal areas (bathrooms, dining room and recreation). Observations were made before and after the intervention. Documentation of non-participant observation data consisted of field notes recorded in field notebooks. These data are records of what the researcher experiences, what he or she learns through interaction with other people, and what the person observes [23].

During the observations, the researchers engaged in informal conversations and/or interviews with the participants. These were informal, unplanned, and unprepared conversations that arose during the work and interaction in the field and study environment that allowed them to discuss and deepen their knowledge of relevant issues, or ask questions about events [23,24]. The interviewer approached the participants and asked them about the possibility of inquiring about their perspectives on aquatic therapy by conducting an informal interview with those who agreed to participate (Table 2). This information was different from information collected using other data collection tools (observation, interviews, and focus groups) [19].

#### 2.7.2. In-Depth Interviews and Focus Groups

The interviews and focus groups were conducted by a researcher who had not been involved in any medical treatment related to the participants. A question guide was used to explore relevant areas of study to address the proposed research objectives. The construction of the question guide was based on previous studies and a literature review [19]. These questions were written with a sufficiently open statement so as not to direct the participants’ answers. The semi-structured interviews and the focus groups were conducted in Spanish, and subsequently audio-recorded and transcribed verbatim after obtaining permission from participants. During the interviews, the researcher took notes on contextual descriptions, participants’ non-verbal responses, the use of metaphors within narratives, and other relevant points raised by the interview participants [19].

### 2.8. Data Analysis

**Figure 1:**
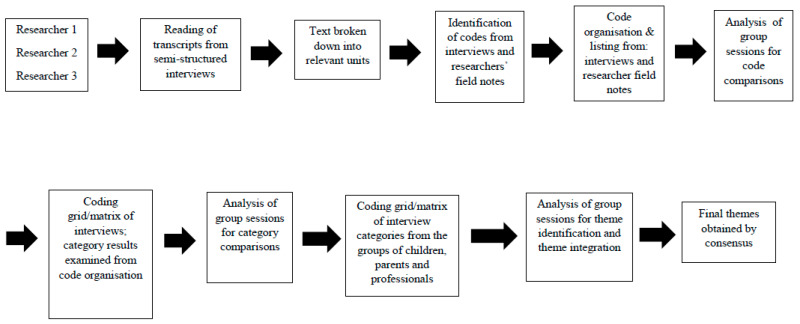
Thematic analysis process.

A thematic and inductive analysis was conducted [19,25] by three researchers (EMB, JGR, and JPC). This type of analysis is consistent with the design in covering the multiple perspectives of the case study participants [20]. Full verbatim transcripts were created for each of the semi-structured interviews, focus groups, informal interviews, and researchers’ field notes [19]. Thematic analysis [25,26] consisted of identifying the most descriptive content to obtain codes and then reduce and identify the most common categories. In this manner, clusters of codes (categories) were formed, i.e., similar points or content that allowed the emergence of themes describing the experience of the study participants [19]. To identify the relevant content, researchers read and reread the text, adding marginal notes and forming initial codes [22]. This method generates an increasing level of abstraction and complexity for the analysis from codes to categories and, finally, themes [19]. This process of coding was performed separately for the interviews and researcher field notes, as well as for the parent and health care professional groups. A coding grid or matrix was created with the codes and categories [27]. A matrix or coding grid is a format used to display qualitative data that systematically presents information so the user can draw conclusions and take needed action(s) [27]. Within this grid, we identified the narratives that justified the obtained results [27]. This process of thematic analysis was carried out separately on non-participant observations, informal interviews, focus groups, and semi-structured interviews. Subsequently, joint meetings were held to pool the results of the analysis. In addition, data collection and analysis procedures were discussed at these meetings. In the event of differences of opinion, the identification of the topic was made via consensus among the members of the research team. Finally, the research team held joint meetings to present, combine, integrate, and identify the final themes [20] (Figure 1). Notably, the final themes were decided by researcher consensus [17,19]. No data analysis software was used.

### 2.9. Quality Criteria

The guidelines for conducting qualitative studies established by the consolidated criteria for reporting qualitative research [28] (http://www.equator-network.org/) and the recommendations for the design of Case Study Research in health care using the DESCARTE model [20] were followed. Also, the criteria for guaranteeing trustworthiness by Guba & Lincoln were applied [29]. The techniques performed and the application procedures used to control trustworthiness are described in Table 3. The use of these methods to increase rigor are compatible with case-study designs [18,30].

### 2.10. Ethics

This study was approved by the Clinical Research Ethics Committee of the San Pablo-CEU University (133/17/TFM) and permission was also obtained from APACE-Talavera. Furthermore, this study adhered to the principles articulated in the WMA Declaration of Helsinki [31]. Written consent and permission to record the interviews were obtained from all of participants and their legal guardians in the case of underage participants.

## 3. Results

Twenty-seven participants (11 observed students, 3 students interviewed, 8 parents participating in focus groups, 3 health professionals, and 2 teachers in interviews) participated in this study. Table 1 displays the sociodemographic data of the participants. The mean age of the children was 10.99 years (SD ± 3.77), the mean duration of school attendance was 5.90 years (SD ± 3.91) and the mean number of years receiving AT was 4.45 years (SD ± 3.20). The most predominant level of the Gross Motor Function Classification System (GMFCS) was V (42.8%). None of the sessions had to be interrupted for safety reasons and none of the children reported adverse effects during the sessions.

The themes representing the participants’ experiences were drawn from semi-structured interviews, focus groups, non-participant observations, and informal interviews. Four themes emerged from the material analyzed: (a) the connection with the environment; (b) postural improvements and mobility; (c) the opportunity to perform tasks; (d) learning and transfer. In the following sections, some of the participants’ narratives are featured, extracted from the interviews or focus groups regarding the four emerging themes [21].

### 3.1. Theme 1—Connection with the Environment

The participants described the numerous, albeit specific, sensory, cognitive, and emotional functions that they felt were ‘awakened’ upon entering a new environment such as the pool, with different mechanical properties, highlighting the possibilities of heightened sensations and a greater connection with the surrounding environment.

“Yes, because for me, water is like my brain, because I feel more alert, I don’t know, it’s like it wakes me up.”

(Student interview)

On several occasions, attention was stated as being the first mental requirement that is activated by the sensory experience of being in the water, favoring the ability for students to perform other activities:

“They pay more attention to conversations as soon as they get into the water, and then they react in the playground when something seems funny or interesting to them, they are more awake than before they get into the water.”

(Therapist interview)

This increased state of alertness ‘awakens’ other emotional functions during the course of the sessions, according to the participants’ descriptions of the feelings of enjoyment and pleasure shown by the children during the treatments in the pool.

“He goes into the water smiling and making sounds. A huge change in behavior and attitude has taken place, compared to how subdued he was. Once in the water, he wants to kiss his therapist while he is being held in the water, breaking into laughter. Afterwards, his face is happy, coupled with an infectious laughter.”

(Informal interview with participant)

For parents, AT offers them a mental and emotional connection with their immediate and close environment, which they also perceive through numerous sensory changes, when they visit other pools outside the school.

“I think they become more mentally active and connected, because, for example, we go down to the pool in the summer and he is more aware of the people around him, who are splashing him, who are making different noises ... he is more attentive to the things that are close to him, more receptive and expressive of how he feels.”

(Parent focus group)

### 3.2. Theme 2—Postural Improvements and Mobility

The participants described the postural improvements and the fluidity of movement perceived during different daily activities, due to the changes they perceived in the children’s muscles.

“It seems that when we dress her to go back to the classroom she has no joint resistance for putting her arms into the shirt sleeves. Once seated in her wheelchair she has good postural control.”

(Informal interviews with participants)

These changes affecting the tissues and postural changes were noticed immediately upon completing the sessions, which they felt could be used to enhance their subsequent performance.

“Their posture is better when they leave and as a result of this better posture they are more willing to do things for their hygiene or clothing. And I think that by moving with less effort in the water, they also improve their posture and their ability to change postures later on.”

(Therapist interview)

The health professionals also described the changes in tone and spasticity together with the greater willingness to collaborate after the sessions.

“Those with more spasticity do come to speech therapy sessions more relaxed when they come from the pool and are more likely to work calmly.”

(Therapist interview)

During the different stages of data collection, professionals and children described the possibilities of movement in water, on a global level.

“Hypertonia is one of the things that makes it very difficult for them, and when they work in water they notice that they move with more fluidity in general, as it is easier for them to move their bodies in the water.”

(Teacher interview)

And sometimes also on a more selective level of movement quality.

“I feel my knees moving, that’s new, I like to feel that they are free.”

(Student interview)

In addition, a recurring theme in the interviews was that, in the water, children experience their greatest potential, as they are not restricted in their ability to move, which motivates them to work harder and autonomously to explore their full capabilities.

“... and in the pool, well if you see him, the other day, walking, in the pool, because he goes with a walker outside, and (in the pool) he walks alone, and he was encouraged to do things by himself.”

(Informal participant interview)

While working in the water, the therapists get a preview of what they will later encounter elsewhere in the school, being able to focus on specific areas of improvement which the children will be able to subsequently apply.

“It is an environment where it is easier to mobilize compared to dry land conditions, so, first you see what the person can do regarding mobility in the water, and then this can be applied in the classroom or the dining room, encouraging us to reinforce this.”

(Interview with therapist)

The participants described how these movement possibilities generate changes, promoting greater independence in transfers and mobility, meaning that children are continuously testing their abilities to move independently.

“She independently climbs the ramp leading outside the pool, sits on the mat, puts on her flip flops, and without any help from anyone, she turns over and gets out of the water.”

(Informal participant interview)

However, participants agreed that subsequent fatigue was a direct consequence of working in the water.

“I end up exhausted, the water lets me move more, and when I finish and get out, if I’ve gone over the top, I can feel it.”

(Student interview)

These are students who experience considerable fatigue due to previous deconditioning, which is why some participants spoke of the need to prescribe aquatic exercise at the appropriate intensity level, so that it does not affect later interventions in the room or classroom.

“Normally they come out feeling more alert, but those who spend more time in their wheelchairs do leave the pool more tired, during the session you can tell that their visual tracking worsens, so we give our own feedback so that the next pool session is lighter.”

(Therapist interview)

The drowsiness induced after working in the water is the main indicator of the effort made during the AT sessions, once again showing teachers that fatigue is a factor that must be controlled.

“That day the overall experience of the parents is that they notice that they return more tired, falling asleep earlier.”

(Teacher interview)

However, the parents say that they appreciate the day their child attends AT, as they sleep better that night, a finding which is unusual because of their typically poor sleeping habits.

“The day at the pool is a comforting sleep... for everyone. He rests and we rest, something so necessary and unusual.”

(Parent focus group)

### 3.3. Theme 3—Possibility of performing Tasks

This theme refers to the moment when the participants carried out educational or therapeutic activities after the sessions in the pool, with greater cognitive, sensorimotor, emotional, and social involvement. The healthcare professionals and educators both spoke of a greater intention to communicate, describing how this affected the children elsewhere throughout the school, by relating more to others, according to their abilities, expressing their opinion and desires to others.

“She is interacting with guttural sounds and making small joyful sounds in class. She is relaxed and attentive. There is smooth, multisensory communication.”

(Informal interviews with participants)

The teachers related how this state of alertness, awakened in the water, influences the communication and writing tasks performed while working in the classroom.

“Although they speak a little louder and interact thanks to the technology of the tablet speaker, they remain concentrated on their work and assignments and are able to write more calmly.”

(Teacher interview)

Likewise, other orofacial functions are facilitated, such as swallowing and phonation.

“Concerning the orofacial work, relaxation and preparation, I notice they are better after going to the pool. In this sense, afterwards, of course, this favors changes in speech, they are able to articulate better. I notice changes in swallowing and feeding on a motor level, which is what we work on the most, together with breathing.”

(Therapist interview)

Regarding swallowing, in specific aspects of feeding, the participants described changes in the children in terms of their hunger and how fast they ate.

“After the pool, they eat the cream of zucchini soup at full speed. They tend to eat very quickly, feeling hungrier when they get out of the water. He waits for the second course by eating bread and chatting with his classmates.”

(Informal interviews with participants)

On numerous occasions, participants described that the experience of working in water meant being able to utilize cognitive functions for other occupational purposes in their daily lives. They initially mentioned the child’s relationship with themselves, referring to changes in self-awareness of their own body, self-determination, and spatial awareness.

“We arrive at the pool and he goes to the mirror and quickly, without hesitation, begins to undress. He does it alone, in front of the mirror, he starts taking off his top and a sneaker, he looks at himself in the mirror, smiles, gets excited by shouting, moderately.”

(Informal participant interviews)

Even in concrete situations regarding self-concept, this was experienced by some children in exceptional ways, regarding the way they perceived their own body.

“...but it had quite a large impact on the first day and she asked her family to please shave her to go to the water, her initiative was great, but it is true that, unfortunately, we don’t have many cases that can have that much of an effect on their own hygiene, given their condition.”

(Teacher interview)

The parents confirmed that this perceived self-concept arising in their children regarding their beliefs and the facilitating environment made their children feel more capable of doing things for themselves.

“When she is in the water playing with other children I see her face that says... ‘I can do it too’, her desire to do participate, and it is not something that they usually express.”

(Parent focus group)

### 3.4. Theme 4—Learning and Transfer

The participants described the most common strategies that children employed in order to participate and engage in active learning processes after the AT sessions. They pointed out how children who were younger or with a greater degree of motor impairment (level V in the GFMCS) or, in some cases, those with less cognitive development used their gaze, listening, attention, and communication skills, which were facilitated after being in the water.

“After getting out of the water she goes to dance class, where she watches the indications given by the teacher from her wheelchair. As it is the older children’s turn to use a walker, she looks at them, leans on her adapted headboard, interacts with the music without losing sight of the dance floor, as if memorizing it, very attentive for how young she is.”

(Informal interview with participant)

Furthermore, they reported how older children, or those with lower levels of impairment (III and IV of GMFCS) or higher cognitive development demonstrated an improved performance of subsequent tasks requiring more advanced thinking processes, such as reading and writing, mathematical processes, or participatory activities and contexts with classroom materials.

“J. when returning to the classroom, he worked better using the tablet for reading and writing. He writes while the teacher spells the word correctly and presses the talkfree device to hear what he has written.”

(Informal participant interview)

All the professionals expressed that the children’s learning was favored, facilitating the subsequent performance of tasks that the children chose, which they preferred to carry out after the aquatic intervention.

All participants emphasized the concept that the swimming pool is a fun and motivating environment which has a positive impact on learning. All the interviews mentioned at some point that the children highly enjoyed the pool, expressing a desire to attend sessions.

“When they go, they are very motivated because they like it very much and they feel like going… they are like: come on, let’s finish up now, let’s do whatever we have to do and let’s go to the swimming pool and have fun.”

(Professional interview)

During the interviews with the children this was increasingly evident, when they were asked what aquatic therapy meant to them, it was not seen as something that they were being forced to do, or being compulsory or laborious, which is often the case of therapies for this population. On the contrary, the participants felt that AT was beneficial for them, allowing them to play, have fun and enabling them to accomplish many things.

“Although we are told that we are going to work a lot in the water, for me it is a game within the school, the fun moment.”

(Student interview)

## 4. Discussion

The findings of this study reveal the perspectives and opinions of children and youth, parents, educators, and therapists regarding the application of AT in the context of special education schools among severely impaired students according to the GMFCS. Our findings provide insight on the relevant practical and meaningful areas of influence that AT targets for the improvement of cognitive and sensorimotor function. The participants in this study also stressed that water is a facilitating environment that promotes participation and transfer of learning.

Our findings cover the main relevant areas of functioning described by the agreed AT CSs for children with neurological disorders [11], with CP being the most prevalent pediatric neurological disorder. The AT CSs project and our school intervention project in CP with severely disabled students agreed on themes related to mental functions, muscle functions, mobility, learning, and transfer. Our results support the idea that the ability to move in the water is an instrumental aspect of AT treatments [11]. Also, the ability for these sessions to enhance subsequent learning and the capacity to task-solve and meet various demands are key features of aquatic interventions, which have a major impact on the school and family environment.

The participants in our study showed that the heightened sensory perception associated with water immersion enables children to experience and awaken functions also on a cognitive level, enabling them to actively connect with their surroundings. In a previous study examining mental functions [14], parents highlighted improvements in concentration, orientation, attention, and emotional processes after beginning AT. The parents justified this because they considered that the children worked in a highly motivating environment, making them feel more awake and alert, as previously outlined by Lai et al. [32]. However, other authors have shown that being in the water changes the cortical activation of the sensory and motor areas, via the hydrostatic pressure offered by the aquatic environment [33,34]. In addition, the site specificity of in-water induced cortical activities has been investigated to assess the activation of sensory, motor, and higher order areas related to motility during water immersion using functional near infrared spectroscopy (fNIRS). Sato et al. demonstrated that water immersion induces cortical activity, and that this activity is specific to specific areas of the brain due to the distribution of neurons activated by specific somatosensory stimulation of the environment [33,34].

In this study, the participants reported changes in the muscles, affecting positioning and mobility, in different situations after coming out of the water. This is consistent with the findings by Chrysagis et al. [35], who described changes in spasticity and active/passive range of motion among children with CP treated with AT. Previous studies have shown that these postural improvements lead to modifications in the motor skills of children with CP receiving AT, with a significant effect on the gross motor function outcomes using the Gross Motor Function Measure (GMFM) [32,36,37]. Lai et al. described such improvements even for children with GMFCS level IV [32]. Similarly, Getz et al. [38] showed that children with more severe motor dysfunction, as classified by the GMFCS, may display superior performance in aquatic environments compared to their performance on land, which is consistent with our findings on the severity of children in special education settings in schools, who are restricted in their ability to perform many activities on land. One possible reason for this may be the thermal and mechanical effects of aquatic exercise [32]. The mechanical properties of the aquatic environment offer benefits by decreasing the effect of gravity and joint loading, optimizing postural control, and muscle strength. The viscosity of the water allows for fluid movement patterns to be experienced. Fragala-Pinkham et al. have argued that these factors improve neuromuscular coordination, muscle endurance and aerobic capacity [39]. In addition, the increased unloading of body weight may facilitate an increase in muscle strength, thus allowing children to initiate movements that are more restricted on land [7,40]. However, in this study, fatigue was mentioned as a potential negative effect on later activities in the classroom or in therapy, suggesting that an appropriate exercise prescription should be made by health professionals, as also noted by Cleary et al. [5].

Our findings reveal that learning and the subsequent transfer of knowledge may be facilitated after working in the water. Thus, Sato et al. [41] argue that in the field of neurological pathology, not all subjects achieve the expected results based on various proposals for rehabilitation at the level of learning and plasticity, due to the variety of pathologies and working environments. However, it should be noted that Sato et al. suggest that while working in water, the effects of neuroplasticity in motor areas of the cortex enhances the performance of essential functions related to motor learning and memory, which are a fundamental part of neurological rehabilitation [41].

Parents of children with CP give importance to communication and its influence on children’s independence, behavior, and relationships [4], in line with our findings regarding communication activities and social participation in schools that promote AT sessions. In the opinion of other authors, however, it is the enjoyment of water activity through play that justifies the higher performance of the children afterwards. Lai et al. [32], showed that aquatic exercises promote pleasure, providing an opportunity to increase children’s enthusiasm and enhancing motor development. Other authors also point to the fact that it is essential for the individual concerned to be cooperative and to participate in the session, to enable appropriate treatment planning and progress [7]. Direct involvement of staff or family members in activities can help encourage children to meet their developmental needs. In particular, during aquatic sessions, the support of parents, teachers, and other health professionals is considered essential. In our study, the main difference with other more clinical settings outlined in AT CSs [11], was the absence of parents during the sessions at school. Although, in this case, family members were not allowed to participate directly during the intervention sessions, throughout the focus group, parents acknowledged that the children demonstrated a positive attitude when going to other pools after receiving AT at the special education school.

This study presents interesting findings in terms of how children perform their tasks or participate in the classroom during their learning processes. Furthermore, the findings regarding the transfer of their basic needs to their daily lives is particularly noteworthy. Thus, the more dependent children enjoyed the moment of grooming, dressing and undressing, and those who were less dependent began to cope more easily with the small challenges of these activities of daily living. The interviews with the professionals revealed that the acquisition of responsibilities through routines, the greater skill in grooming activities, self-respect and self-knowledge, and the dressing and undressing that are developed in relation to the aquatic activity generated further learning and a subsequent transfer of skills. Overall, after the sessions in the pool, they described interesting dynamics in the classroom, intervention rooms at school or at the home, where they pointed out that learning was transferred from the educational/therapeutic environment to the family.

It is important to note that case studies include different units of analysis that allow a more in-depth understanding of the phenomenon, as well as serving as a triangulation technique to enhance the quality of the study and to provide information from other perspectives, besides the student’s own perspective [20,28,30]. Nonetheless, this study has certain limitations. First, the study included a small number of participants. Second, the qualitative nature of this study means that the purpose of this study was to describe the experiences of the participants and therefore these findings cannot be generalized. Finally, the number of female and male participants was different in each group. This may affect findings, as the perspectives may be different based on the participants’ gender.

## 5. Conclusions

Our findings may help to better understand the benefits of different intervention approaches for children with severe CP in special education settings. Thus, this study highlights the use of AT, as an alternative treatment approach which can be applied in these schools. The children and parents of this study felt that these AT sessions were helpful, making the children feel happy, relaxed, and calm, as well as enabling them to participate in further activities for the remainder of the school day. For the professionals, AT is motivating, enjoyable, and beneficial for the health of children and youth, enabling them to further explore their capabilities. The different mechanical properties offered by water favor learning and encourage the ability for children to participate in their educational, therapeutic, and family environment. These results can be used to develop and coordinate intervention programs in controlled environments, such as specialist school settings. These experiences may also be applied to other types of pathologies that affect children and youth, other than CP, as well as involving the family members of children on these programs.

## Figures and Tables

**Table 1:** Sociodemographic data of participants

Participants	Sociodemographic Data
Students	Participants: 14 (5 women)
	Mean age (years): 10.90 (SD +/− 3.77)
	School attendance (years): 5.90 (SD +/− 3.91)
	Number of years receiving aquatic therapy: 4.45 (SD +/− 3.20)
	% GMFCS: II (28.5%), III (14.2%), IV (14.2%), V (42.8%)
Parents	Participants: 8 (7 women)
	Mean age: 46 (SD +/− 6)
Health care professionals	Participants: 3 (3 women)
	Mean age: 39 (SD +/− 3.5)
	Years of experience in CP: 12 (SD +/− 4.96)
Education professionals	Participants: 2 (2 women)
	Mean age: 36.5 (SD +/− 0.5)
	Years of experience in CP: 14.5 (SD +/− 2.5)

**Table 2:** Data collection process

Participants	Data Collection Tool	Number of Participants	Setting	Time	Study Phase
Children context					
Children	Participant observation	11 students	APACE School (Classrooms, hall, playground, dining room, speech therapy room, pool, dressing room, occupational therapy room, bathroom, dancing activity → hall, weaving workshop → classroom, physiotherapy room, corridors, social skills workshop with psychotherapist → classroom, storyteller activity → classroom)	59 h (3540 min)	During period of intervention (October/June)
				Minimum 40 min before aquatic intervention + minimum 40 min after aquatic intervention	
	Semi-structured interviews	3 students	APACE Classroom	210 min	During intervention, after school
				(range: 63 to 77 min)	
Parents	Focus group	8 parents	APACE School dining room	73 min	During intervention, after school
Therapy context	Semi-structured interviews	3 health professionals	Assistant → APACE Classroom	249 min	During intervention, during breaks after school
			Speech therapist → APACE Speech therapy room		
			Physiotherapist → APACE Physiotherapy room	(range: 77 to 92 min)	
	Informal interviews	2 health professionals	APACE school	40 min	During period of intervention
Educational context	Semi-structured interviews	2 teachers	Director → APACE Classroom	123 min	During intervention, after school
			Teacher → APACE Classroom	(range: 55 to 68 min)	
	Informal interviews	1 teacher	APACE school	22 min	During period of intervention

**Table 3:** Trustworthiness criteria

Criteria	Techniques Performed and Application Procedures
Credibility	Investigator triangulation: each interview was analyzed by two researchers. Thereafter, team meetings were performed in which the analyses were compared and themes were identified.
	Triangulation of data collection methods: interviews were conducted, and researcher field notes were kept.
	Participant validation: this consisted of asking the participants to confirm the data obtained during the data collection and analysis stages.
Transferability	In-depth descriptions of the study were performed, providing details of the characteristics of researchers, participants, contexts, sampling strategies, and the data collection and analysis procedures.
Dependability	Audit by an external researcher: an external researcher assessed the study research protocol, focusing on aspects concerning the methods applied and study design.
Confirmability	Investigator triangulation, participant triangulation, data collection triangulation.
	Researcher reflexivity was encouraged via the completion of reflexive reports and by describing the rationale for the study.

## Supplementary Materials

The following are available online at https://www.mdpi.com/1660-4601/17/10/3690/s1, Table S1: Questions for children; Table S2: Questions for parents; Table S3: Questions for professionals.
