# Supplementary material for: Influence of Aquatic Therapy in Children and Youth with Cerebral Palsy: A Qualitative Case Study in a Special Education School
Source: Int J Environ Res Public Health. 2020 May 23;17(10):3690. doi: 10.3390/ijerph17103690 (PMC7277651; doi:10.3390/ijerph17103690)
Supplement: Supplementary file 1 [file ijerph-17-03690-s001.pdf]

Supplemental material S1. Semi-structured interview guide questions for children

Research areas	Questions
Meaning of aquatic therapy and overall experience	How has your experience been with the aquatic therapy program at the school?
Aquatic therapy treatment	What do you feel is most important in this treatment?
Influence of aquatic therapy on function, structures, activity & participation	How is your body after being in the water? How is your movement?
Factors for which aquatic therapy is most useful	What do you like the most about doing therapy in water?

Supplemental material S2. Focus group guide questions for parents

Research areas	Questions
Meaning of aquatic therapy and prior experiences	What does it mean to you that your child receives aquatic therapy sessions at school?
Aquatic therapy treatment	What do you consider has been the most relevant aspect of this treatment?
Influence of aquatic therapy on the daily care and management of the child	How is your child the day he/she goes to the pool?
Factors for which aquatic therapy is most useful	How do you think aquatic therapy helps to improve your child's health?
	What do you think aquatic therapy can provide you in the long term?

Supplemental material S3. Semi-structured interview guide questions for professionals

Research areas	Questions
Meaning of aquatic therapy and prior experiences	What do you think of aquatic therapy as an intervention within the school?
Aquatic therapy treatment	What do you consider has been the most relevant aspect of this treatment?
Influence of aquatic therapy on the daily care and management of the child in school	What feelings does the child convey the day that you know he/she has been in the pool?
Factors for which aquatic therapy is most useful	What do you think aquatic therapy brings to his/her life?<br>How do you feel aquatic therapy can affect him/her in the long term?
